# piR-hsa-211106 Inhibits the Progression of Lung Adenocarcinoma Through Pyruvate Carboxylase and Enhances Chemotherapy Sensitivity

**DOI:** 10.3389/fonc.2021.651915

**Published:** 2021-06-23

**Authors:** Yongmei Liu, Yanhan Dong, Xinjia He, Anjing Gong, Jinning Gao, Xiaodan Hao, Shuai Wang, Yuqiao Fan, Zibo Wang, Meng Li, Wenhua Xu

**Affiliations:** ^1^ Department of Inspection, The Medical Faculty of Qingdao University, Qingdao, China; ^2^ Institute of Translational Medicine, Qingdao University, Qingdao, China; ^3^ Department of Radiation Oncology, The Affiliated Hospital of Medical College Qingdao University, Qingdao, China; ^4^ Department of Neurosurgery, The Affiliated Hospital of Qingdao University, Qingdao, China

**Keywords:** piRNA, lung adenocarcinoma, cancer, non-coding RNA, pyruvate carboxylase, drug sensitivity

## Abstract

Although the importance of PIWI-interacting RNAs (piRNAs) in cancer has recently been recognized, studies on the role and functional mechanism of piRNAs in lung adenocarcinoma (LUAD) development and progression are limited. In this study, we identified 10 differently expressed piRNAs in LUAD tissues compared to normal tissues, among which, piR-hsa-211106 expression levels were downregulated in LUAD tissues and cell lines. Furthermore, the effects of piR-hsa-211106 on the malignant phenotypes and chemosensitivity of LUAD cells were detected by gain- and loss-of-function analyses *in vitro* and *in vivo*, which showed that piR-hsa-211106 inhibited LUAD cell proliferation, tumor growth, and migration, but promoted apoptosis. Moreover, our finding indicated that piR-hsa-211106 is a potential therapeutic target that synergistically imparts anticancer effects with a chemotherapeutic agent for LUAD-cisplatin. Further mechanistic investigation indicated that piR-hsa-211106 could bind to pyruvate carboxylase (PC) by RNA pull down and RNA immunoprecipitation assays and inhibited *PC* mRNA and protein expression. Our study demonstrates that piR-hsa-211106 inhibits LUAD progression by hindering the expression and function of PC and enhances chemotherapy sensitivity, suggesting that piR-hsa-211106 is a novel diagnostic and therapeutic target for LUAD.

## Introduction

Lung cancer is one of the most common malignancies and the major cause of cancer-related death worldwide (1). Lung adenocarcinoma (LUAD) is the most common subtype of lung cancer, accounting for 40% of diagnoses (2, 3). Although advances in surgery, radiotherapy, and systemic treatment have significantly improved the clinical outcome of patients with LUAD, the average 5-year survival rate only achieves 15% of cases (4–6). Early detection and early treatment are key factors to improve the survival rate of LUAD patients (7, 8). Therefore, there is an urgent need to clarify the potential mechanisms of tumorigenicity and metastasis, which can provide better treatment strategies for LUAD.

Cancer cells undergo many metabolic reprogramming and produce a variety of metabolic adaptations, which are associated with cell proliferation, survival, and metastatic progression (9). The Warburg effect indicates that cancer cells will continue to utilize glycolysis for energy production even in the presence of abundant oxygen and have the ability to alternate between the use of glycolysis or oxidative phosphorylation (OXPHOS) for energy production in response to particular environmental stresses (10). The axis of pyruvate utilization is a key regulatory point in cancer cell metabolism, which is critical in lung metastatic deriving from breast cancer (11). Pyruvate carboxylase (PC) is a major anaplerotic enzyme for the carboxylation of pyruvate to oxaloacetate *via* the ATP-dependent pathway in the Krebs cycle and is also a key enzyme in gluconeogenesis and *de novo* fatty acid and amino acid synthesis in normal cells (12). According to reports, PC is overexpressed in non-small cell lung cancer (NSCLC) tissues compared with normal tissues, meeting the increased anabolic and energetic demands of cancer cell proliferation (13). However, the upstream regulatory mechanism of PC in lung cancer is unknown.

In the past decade, non-coding RNAs (ncRNAs), such as microRNAs (miRNAs) and long non-coding RNAs (lncRNAs), have attracted considerable attention in cancer research because they regulate gene expression and functionally interact with other molecules, significantly contributing to cancer pathogenesis. Recently, more and more data from RNA-sequencing of cancer specimens have led to the discovery of additional classes of novel ncRNAs such as PIWI-interacting RNAs (piRNAs), which is an important emerging participant in cancer pathogenesis (14). piRNAs were first discovered in 2001 in *Drosophila* testes as small RNAs derived from the Su(Ste) tandem repeats, which silence Stellate transcripts to maintain male fertility (15). To date, more than 20,000 piRNAs have been identified in germ and somatic cells (16, 17). piRNA comprise by 24 to 31 nucleotides (nt) with a 5′-terminal uridine or 10^th^-position adenosine bias, lacking clear secondary structure motifs (18). The multifaceted functions of piRNAs have been demonstrated (19–21). piRNAs can bind to piwi proteins to form a piRNA/piwi complex that influences transposon silencing, spermiogenesis, genome rearrangement, epigenetic regulation, protein regulation, and germ stem-cell maintenance (22). Although little is known about the functions of piRNAs in human cancer, aberrant piRNA expression in some cancers indicates that piRNAs may play an important role in tumorigenesis and are associated with cancer prognosis (23, 24).

Here, we report the identification of piR-hsa-211106 that is significantly downregulated in LUAD tissues compared with normal tissues and acts as a possible cancer suppressor. We elucidated the molecular mechanism for the function of piR-hsa-211106 in LUAD cells. Furthermore, we have also found that upregulated expression of piR-hsa-211106 imparts a synergistic anti-cancer effect with chemotherapeutic agents on LUAD cells.

## Materials and Methods

### Clinical Specimens

We collected 20 pairs of cancerous and normal tissues (at least 5 cm from the tumor) of LUAD patients who underwent lobectomy in the Affiliated Hospital of Qingdao University (Shandong, China) from 2018 to 2019. All LUAD patients were diagnosed by histopathology, and the tumor staging was based on the 7th edition of the AJCC Cancer Staging System. All tissue samples were collected during the operation. The tissue samples were immediately frozen and stored in liquid nitrogen until analysis.

### piRNA Sequencing and Selection

PiRNA sequencing of LUAD tissues and normal lung tissues was completed by Beijing Biomark Biotechnology Co., Ltd. The sequencing results were submitted to BLAST on the NCBI website, and the result files were screened according to the criteria of “unique gene locus”, “length between 24 and 31 nucleotides”, and “base T at the 5′ end” to obtain high quality piRNA data. Only tumor samples with piRNA counts per million mapped reads (CPM) ≥ 1 and expression above 20% are considered fully expressed and can be further analyzed. In this study, we selected the 10 differentially expressed piRNAs for research (five upregulated and five downregulated).

### Cell Lines and Cell Culture

Human A549 and HCC2279 LUAD cell were donated by Professor Yu Zhuang’s research group from the Department of Oncology, the Affiliated Hospital of Qingdao University (Shandong, China). A549 and HCC2279 cells were maintained in DMEM (Gibco, USA) medium supplemented with 10% fetal bovine serum (TransSerum FQ Fetal Bovine Serum, China) and 1% antibiotics (Meilunebio, China). All cell lines were grown in a 37°C atmosphere with 5% CO_2_ and 99% relative humidity. The cell lines were passaged for less than 6 months. No mycoplasma infection was found in any cell line.

### RNA Extraction and Quantitative Real-Time PCR Analysis (qRT-PCR)

TRIzol reagent (Invitrogen, USA) was used to extract total RNA from cell lines and clinical tissues. A Revert Aid First Stand cDNA Synthesis Kit (Takara, Japan), random primers, specific piRNA reverse transcription primers were used to synthesize first-strand cDNA. RNA level was determined in triplicate by qRT-PCR on CFX-96 using the SYBR Green method. The comparative Ct method was used to normalize RNA levels to GAPDH RNA, and piR-hsa-211106 levels to U6. The primer sequences used in this study were described in **Table 1**.

**Table 1:** The primer sequences used in this study.

Gene	Primer sequence
**piR-hsa-211106**	RT: 5′-GTCGTATCCAGTGCAGGGTCCGAGGTATTCGCACTGGATACGACAAAATT-3′
	Forward: 5′-GGCTCGAAGGACTTCGTCTGT-3′
	Reverse: 5′-AGTGCAGGGTCCGAGGTATT-3′
**U6**	Forward: 5′- CTCGCTTCGGCAGCACA -3′
	Reverse: 5′- AACGCTTCACGAATTTGCGT -3′
**miR-3180-3P**	RT: 5′-GTCGTATCCAGTGCAGGGTCCGAGGTATTCGCACTGGATACGACGGCCTC -3′
	Forward: 5′-TGGGGCGGAGCTTCCG-3′
	Reverse: 5′-AGTGCAGGGTCCGAGGTATT- -3′
**PC**	Forward: 5′-GCTGGAGGAGAATTACACCCG-3′
	Reverse: 5′-GGATGTTCCCATACTGGTCCC-3′
**GAPDH**	Forward: 5′- ACCACAGTCCATGCCATCAC -3′
	Reverse: 5′- TCCACCACCCTGTTGCTGTA -3′
**3**′**-RNA adapter**	5′- UCGUAUGCCGUCUUCUGCUUGU -3′
**Methyl-anchored**	RT: 5′-GGACGGATGGAGGTGCAGCTACACAAGCAGAAGACGGCATACGAA -3′
**Methyl-unanchored**	RT: 5′-GGACGGATGGAGGTGCAGCTACACAAGCAGAAGACGGCATACGA-3′

### Fluorescence *In Situ* Hybridization (FISH)

FISH analysis was performed to localize piR-hsa-211106 in LUAD cells. The cells were seeded in a 48-well plate at a density of 1 × 10^4^ cells/well and cultured overnight in an incubator. The next day, 4 μM Cy3-labeled piR-hsa-211106 fluorescent probe (GenePharma, China) was hybridized to the cells by incubating in a 37°C incubator overnight in the dark. The following day of hybridization, the fluorescent probe was aspirated and discarded, and DAPI working solution was added to stain the cell nuclear by incubation for 20 min in the dark, followed by observation under a fluorescent microscope. Cy3-labeled piR-hsa-211106 fluorescent probe sequence was described in **Table 2**.

**Table 2:** Sequence of transfection.

Gene	Sequence
**piR-hsa-211106 -agomir**	Sense: 5′-UCCGGCUCGAAGGACUUCGUCUGUAAUUUU-3′
	Antisense: 5′-AAUUACAGACGAAGUCCUUCGAGCCGGAUU-3′
**agomir -NC**	Sense: 5′-AUUCUUCAUGUUCCUACGUAGACGUGGCGU-3′
	Antisense: 5′-GCCACGUCUACGUAGGAACAUGAAGAAUUU-3′
**piR-hsa-211106-antagomir**	5′-AAAAUUACAGACGAAGUCCUUCGAGCCGGA-3′
**antagomir-NC**	5′-UCAUUUUGUGUAGUACAA-3′
**Bio-piR-hsa-211106**	5′-UCCGGCUCGAAGGACUUCGUCUGUAAUUUU-3′
**Cy3-piR- probe**	5′-AAAATTACAGACGAAGTCCTTCGAGCCGGA-3′

### RTL-P Analysis of piRNA 2′-O-Methylation

To detect the 2′-O-methylation of piR-hsa-211106, modified RT-PCR conducted as previously described (25). Reverse transcription (RT) was performed in 25-μl reaction mixtures, each containing 100 μg total RNA and 50 μM specific RT primers, denatured at 70°C for 10 min and then placed on ice. The RT buffer, 200 µU M-MLV reverse transcriptase (Takara, Japan), 40 µU RNasin ribonuclease inhibitor (Takara, Japan) and a low (0.4 μM) or high (40 μM) concentration of dNTP (Takara, Japan) were mixed, initial annealing was performed at 42°C for 1 h, followed by heating at 70°C for 15 min. The PCR reaction was then determined, and the PCR products were separated on a 2% agarose gel and visualized by UV-transmitted illumination.

### Oligonucleotide Transfection

A549 and HCC2279 cells were seeded in six-well plates and cultured to 60% to 70% confluency before transfection. The cells were transfected with piR-hsa-211106 agomir, piR-hsa-211106 inhibitor, and negative control oligonucleotide (GenePharma, China) with the help of Lipofectamine™ 3000 reagent (Invitrogen, USA). The transfection efficiency was verified by qRT-PCR. The sequence of agomir, inhibitor and NC were described in **Table 2**.

### Lentivirus Production and Transduction

To construct a recombinant lentiviral vector expressing piR-hsa-211106, the precursor of piR-hsa-211106 was inserted downstream of the CMV promoter in pLent-Puro-GFP (GenePharma, China), and an empty pLent-Puro-GFP vector was employed for comparison. A short hairpin RNA (shRNA) specifically targeting piR-hsa-211106 was synthesized and subcloned into the pSIH1-Puro-GFP lentiviral shRNA vector (GenePharma, China). HEK293T cells were transfected with a vector and lentiviral vector packaging system to produce lentiviruses. In the presence of polybrene (GenePharma, China) A549 cells were infected with the corresponding concentrated lentivirus and screened with puromycin hydrochloride to establish a cell line with stable overexpression or knockdown of piR-hsa-211106.

### Cell Viability Determination

For cell viability analysis, A549 and HCC2279 cells (1 × 10^3^ per well) were seeded in 96-well plates. After culturing for a certain period of time, the cell viability was measured with a CCK-8 kit (Yeasen, China).

### Immunofluorescence Assay

The cells (1.5 × 10^3^ to 1.5 × 10^4^ per well) were seeded in a 96-well plate. After culturing for 48 h, the cells were labeled with 30 μmol/L 5-ethynyl-2′-deoxyuridine (EdU) for 2 h. After staining with 4% paraformaldehyde for 30 min, staining was conducted with Hoechst (Yeasen, China), and photographs were taken under a fluorescence microscope.

### *In Vitro* Migration Assay

The migration assay was performed in a 24-well Millicell chamber. Approximately 200 μl of cells (2 × 10^4^) in serum-free medium were added to the coated filter membrane, and then 500 μl of medium containing 20% fetal bovine serum was used as a chemoattractant and added to the lower chamber. After incubating at a 37°C for 48 h, cells that migrated through the filter were fixed with 4% paraformaldehyde and stained with 0.5% crystal violet, and the number of cells was counted in three random fields.

### Wound Healing Experiment

LUAD cells were cultured in a six-well plate, and the cell monolayer was later scraped with a 200-μl pipette tip. A representative image of cell migration was captured by images taken at 10× high power magnification 0 and 24 h after scratching. The reduced distance across the induced damage area was measured, normalized to the 0 h control, and expressed as the relative migration rate.

### Apoptosis Analysis

Cell apoptosis analysis was performed using an Annexin V-FITC Cell Apoptosis Detection Kit (Yeasen, China), following the manufacturer’s recommendations. The percentage of apoptotic cells was determined by flow cytometry (BD Accuri C6).

### Biotin-Coupled Probe RNA Pull-Down Experiment

Approximately 1 × 10^7^ LUAD cells were harvested, lysed, and sonicated. The biotinylated piR-hsa-211106 (GenePharma, China) probe were incubated with the probe-M280 streptavidin Sepharose beads (Invitrogen, USA) at 25°C for 2 h to generate probe-coated beads. The cell lysate and probe-coated bead mixture was then incubated overnight at 4°C. The next day, the mixture was washed with buffer, and the RNA complex bound to the beads was eluted and purified with TRIzol reagent (Takara, Japan) for further analysis.

### Liquid Chromatography-Tandem Mass Spectrometry (LC–MS/MS)

Proteins pulled down by piR-hsa-211106 in SDS-PAGE were analyzed by Shanghai Zhongke New Life Biotechnology Co., Ltd. The raw file of mass spectrometry test was used to search the corresponding database with Mascot2.2 software, and finally the result of the identified protein was obtained. The results were then analyzed by BLAST on the GO database, and the result files were screened using the criteria “PepCount≥10” and “UniquePepCount≥10” to obtain high-quality protein data. Only proteins that were considered functional in the GO database were further analyzed. In this study, we selected seven proteins for investigation.

### RNA Immunoprecipitation (RIP)

RIP assay was performed using the RIP Kit (Geneseed, China). Antibodies PC (Proteintech, China) and IgG (Abcam, China) was used. RNA (PC binding) and isotype control (IgG binding) for each antibody were assayed simultaneously. The co-precipitated RNAs were detected by qRT-PCR.

### Western Blot

Protease inhibitors (Meilunebio, China) were added into the radioimmunoprecipitation assay lysis buffer (RIPA, Meilunebio, China) and used to lyse cells for protein extraction. The protein concentration was determined by bicinchoninic acid (Solarbio, China). The same amount of protein was analyzed by 10% SDS-PAGE and transferred it to a polyvinylidene fluoride membrane (PVDF, Roche, Switzerland). The membrane was blocked with 5% skimmed milk powder, followed by the primary antibody PC (1:5,000, Proteintech, China), anti-GAPDH (1:10,000, CST, USA) with 5% bovine serum albumin (Solarbio, China), and incubated overnight at 4°C. Then, the prepared membrane was incubated with the secondary antibody (1:10,000, Abcam, China) for 2 h. Finally, an ECL chemiluminescence reagent (Vazyme, China) was employed to observe the membranes, and relevant data were analyzed using Quantity One software.

### Chemical Sensitivity Check

Cells were seeded into a 96-well plate (1 × 10^3^ cells per well), and a series of concentrations of cisplatin was added to the medium the next day. After 96 h of incubation, the cell viability was determined using a CCK8 kit. The drug concentration that reduces cell viability compared with the control cells was calculated. Subsequently, cells with stably knocked down or enforced piR-hsa-211106 were co-cultured with cisplatin at concentrations of 20 and 40 µM to determine whether the synergy or antagonism occurred between piR-hsa-211106 and cisplatin, respectively.

### Animal Experiment

BALB/c nude mice aged 3 to 4 weeks were purchased from Beijing (Biotechnology Co., Ltd), and used for experiments after 1 week of acclimatization. To determine the effect of piR-hsa-211106 on the growth of implanted tumors, a total of 2 × 10^7^ A549 cells with overexpressed or knocked down piR-hsa-211106 were injected subcutaneously into the armpits of mice (five mice per group). When the tumor was palpable, as measured every 7 days, its volume was calculated using the following equation = Length × Width^2^ × 0.5. The experiment ended four weeks after tumor implantation. The Living Image^®^ system (Perkin Elmer) was employed to detect lesions.

Groups of animals with subcutaneous xenograft were also treated with chemotherapeutic agents to test the effects of piR-hsa-211106 on chemosensitivity of LUAD cells. Cisplatin (5 mg/kg body weight/week) was intraperitoneally injected to mice (five per group) when the xenograft reached 50 mm^3^. We also treated the subcutaneous xenograft with agopiR-hsa-211106 (GenePharma, China) by intratumor injection to determine the effect of piR-hsa-211106. Briefly, when the tumors implanted on the left and right back sides of the mice reached a certain volume, 50 µM agopiR-hsa-211106 was directly injected into the tumor on the right side. AgopiR-NC (GenePharma, China) was injected into the tumor at another site as a control. Treatment was performed every other day for 28 days, and the tumor volume was measured once a week. At the end of the experiment, the mice were sacrificed, and the xenografts were peeled off and photographed. The experimenters turned a blind eye to the cells, drugs, and solvents injected into the mice. All experimental procedures were conducted in accordance with approved protocols and guidelines from the Institutional Animal Care and Use Committee of Qingdao University.

### Statistical Analysis

Statistical analysis was performed using SPSS 22.0 software (SPSS, Chicago, IL, USA) and GraphPad Prism 8.0 (GraphPad Software, Inc., USA). The results are presented as mean ± SEM. Comparison between two groups was assessed using Student’s *t* test (two-tailed, with P<0.05 considered significant).

## Results

### piR-hsa-211106 Is Downregulated in LUAD Tissues and Different Cancer Cell Lines

**Figure 1:**
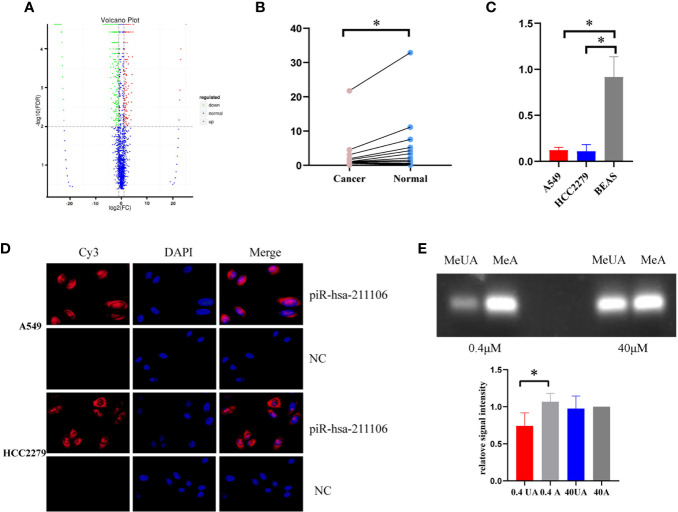
piR-hsa-211106 is downregulated in LUAD tissues and cells. **(A)** The high-throughput piRNA sequencing volcano map shows that compared with normal tissues, there are 1,333 downregulated piRNAs and 236 upregulated piRNAs in LUAD tissues; **(B)** The expression level of piR-hsa-211106 in LUAD tissues and matched non-tumor tissues (mean ± SEM; *p < 0.05); **(C)** The expression level of piR-hsa-211106 in A549 and HCC2279 LUAD cells and BEAS normal lung cells (mean ± SEM; *p < 0.05); **(D)** Fluorescence *in-situ* hybridization assay shows that piR-hsa-211106 is located in the cytoplasm and nucleus. DAPI stains the nucleus in blue, and the probe label of piR-hsa-211106 is labeled with Cy3 and stains red; **(E)** 3′ end methylation detection of piR-hsa-211106 through RTL-P analysis in A549 cells. Representative images (upper) and quantitative statistics (lower) of RTL-P analysis. All experiments were repeated three times. LUAD, lung adenocarcinoma; MeA, methyl-anchor; MeUA, methyl-unanchor.

High-throughput sequencing of piRNAs from LUAD tissues and its matched normal tissues was performed (**Figure 1A**). We screened five upregulated and five downregulated piRNAs according to requirements described in the *Materials and Methods* section. qRT-PCR analysis was conducted to validate the expression levels of the above 10 piRNAs in a set of 20 LUAD tissues and its matched normal tissue samples. Compared with normal tissues, piR-hsa-211106 was stably reduced in tumor tissues (**Figure 1B**). This was subsequently confirmed in different A549 and HCC2279 LUAD cells (**Figure 1C**). In addition, FISH analysis showed that piR-hsa-211106 was distributed in both the nucleus and cytoplasm (**Figure 1D**). Based on the characteristics of piRNAs, we also applied RTL-P assay to confirm methylation of the 3′ end of piR-hsa-211106 in A549 cells (**Figure 1E**).

### piR-hsa-211106 Inhibits the Proliferation and Promotes the Apoptosis of LUAD Cells *In Vitro*

**Figure 2:**
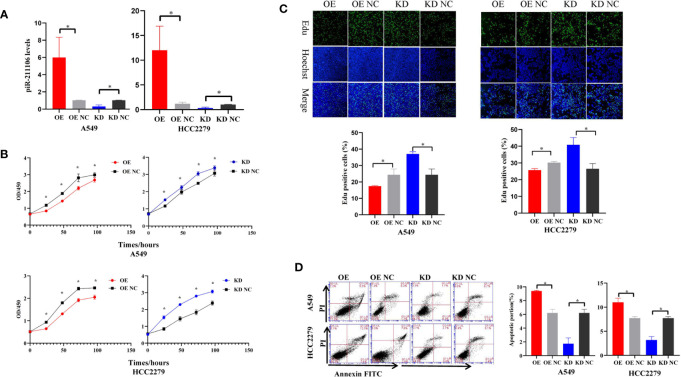
piR-hsa-211106 inhibits the proliferation and promotes the apoptosis of LUAD cells. **(A)** Transfection efficiency of piR-hsa-211106 agomir and antagomir transfected in A549 and HCC2279 cells (mean ± SEM; *p < 0.05); **(B)** Effect of piR-hsa-211106 expression on A549 and HCC2279 cell proliferation tested by CCK8 assay (mean ± SEM; *p < 0.05); **(C)** The fluorescent thymidine analog EdU was used to identify proliferative cells by labeling their DNA (green signal). Nuclei labeled with Hoechst are presented in blue. Representative images (upper) and quantitative statistics (lower) (mean ± SEM, *p < 0.05); **(D)** Effect of piR-hsa-211106 on A549 and HCC2279 cell apoptosis tested by flow cytometry. Representative images (left) and quantitative statistics (right) (mean ± SEM, *p < 0.05). All experiments were repeated three times. OE, overexpression; OE NC, overexpression negative control; KD, knock down; KD NC, knock down negative control.

Because piR-hsa-211106 was dramatically reduced in LUAD tissues and cells, we want to determine whether affect LUAD cell growth. We first synthesized agomir and antagomir that could overexpress or knock down piR-hsa-211106 *in vitro* and then verified the efficiency of overexpression and knockdown in A549 and HCC2279 cells after transfection with agomir and antagomir, respectively (**Figure 2A**). We found that overexpression of piR-hsa-211106 significantly attenuated A549 and HCC2279 cell proliferation, while piR-hsa-211106 suppression dramatically facilitated cell proliferation as indicated by the CCK8 and EdU incorporation assays (**Figures 2B, C**). Flow cytometry analysis have showed that A549 and HCC2279 cells with enhanced piR-hsa-211106 exhibited a significant increase in apoptosis, while knocking down notably apoptosis significantly (**Figure 2D**).

### piR-hsa-211106 Attenuates the Migration of LUAD Cells *In Vitro*

**Figure 3:**
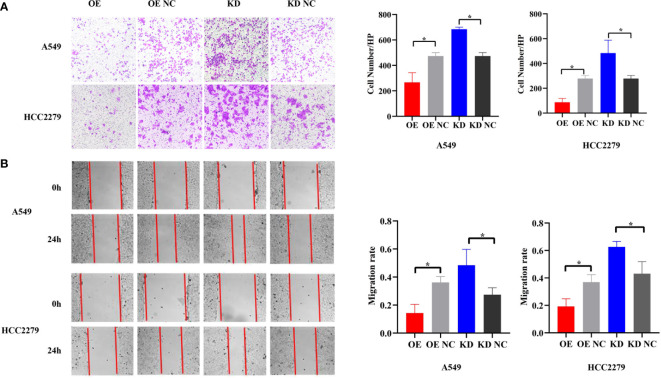
piR-hsa-211106 inhibits the migration of LUAD cells. **(A, B)** Effect of piR-hsa-211106 on the migration of A549 and HCC2279 LUAD cells *in vitro*. Representative images (left) and quantitative statistics (right) of Transwell analysis **(A)** and wound-healing assay **(B)** (mean ± SEM, *p < 0.05). All experiments were repeated three times.

Because piR-hsa-211106 affected the growth of LUAD cells *in vitro*, we then determined whether it could inhibit the migration ability of LUAD cells. Transwell assay showed that, compared with the control, enforced piR-hsa-211106 suppressed the migration ability of A549 and HCC2279 cells; the number of cells passing through the chamber was low, whereas knocking down piR-hsa-211106 enhanced the migration capacity of A549 and HCC2279 cells, as indicated by a higher rate of passing through the chamber (**Figure 3A**). The results of the wound-healing assay coincided with those the Transwell assay. Overexpression of piR-hsa-211106 attenuated the centripetal migration of LUAD cells, whereas piR-hsa-211106 suppression accelerated this activity (**Figure 3B**).

### piR-hsa-211106 Controls the Growth of Nude Mice Xenografts *In Vivo*

**Figure 4:**
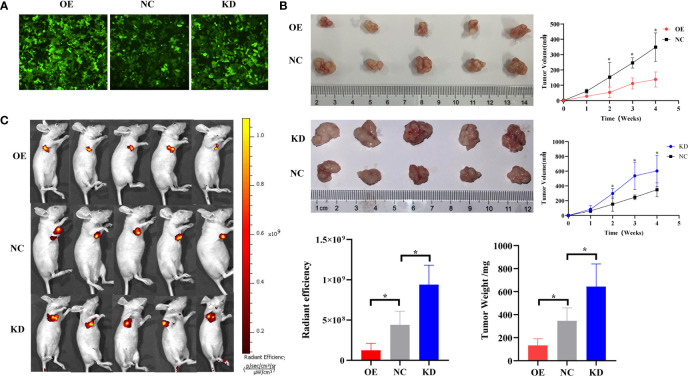
piR-hsa-211106 inhibits tumor growth *in vivo*. **(A)** Construction of A549 cells with piR-hsa-211106 stably overexpressed or knocked down; **(B)** Effect of piR-hsa-211106 on the growth of subcutaneous xenografts in nude mice. Representative images (left) and quantitative statistics (right and bottom right) (mean ± SEM, *p < 0.05); **(C)** Live imaging of a nude mouse tumor. Luminescence imaging of tumor (left) and quantification (right) (mean ± SEM, *p < 0.05).

We have confirmed that piR-hsa-211106 can affect the malignant phenotype of LUAD cells *in vitro.* To further verify whether piR-hsa-211106 can inhibit the growth of nude mouse xenografts *in vivo*, we transfected A549 cells with lentivirus and constructed stable transfected cell line A549 with piR-hsa-211106 stably overexpressed or knocked down piR-hsa-211106 (**Figure 4A**). Afterward, the stable transfected A549 cells were injected into the armpits of tumor-bearing mice. Four weeks after the tumors were palpable, the nude mice were sacrificed, and the tumors were stripped. We observed that the xenografts of the overexpression group were significantly smaller than those the control, while the volume of the knockdown group was significantly larger (**Figure 4B**). Since the second week of volume measurement, the volume of the xenografts in overexpression and knockdown groups began to show statistically significant differences. Before sacrifice, the nude mice were anesthetized with 10% chloral hydrate for live imaging. We found that compared with the control, the fluorescence range of the overexpression group was small, and the fluorescence range of the knockdown group was large (**Figure 4C**). The above results indicate that piR-hsa-211106 might slow down the growth of nude mouse xenografts *in vivo*.

### Pyruvate Carboxylase Is a Downstream Target of piR-hsa-211106

**Figure 5:**
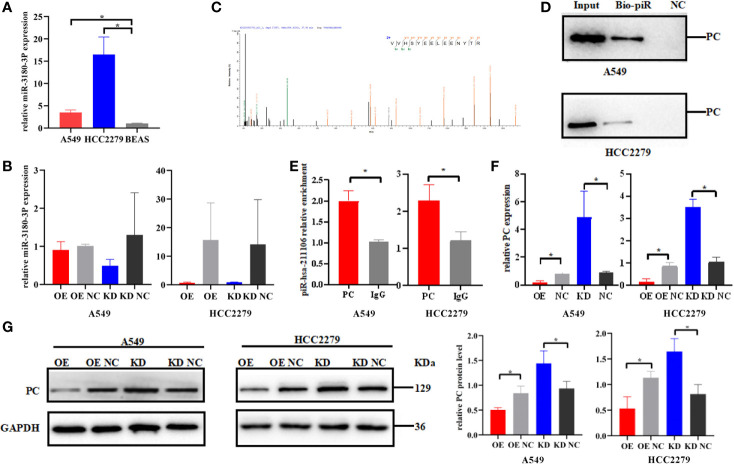
Pyruvate carboxylase is the downstream target of piR-hsa-211106. **(A)** The expression level of miR-3180-3P in A549 and HCC2279 LUAD cells and BEAS normal lung cells (mean ± SEM; *p < 0.05). **(B)** Effect of piR-hsa-211106 on miR-3180-3P (mean ± SEM; *p* > 0.05). **(C)** One of peptides of PC protein identified by LC -MS/MS is represented; **(D, E)** piR-hsa-211106 can interact with PC in LUAD cells tested by RNA pull down and RIP assays; **(F)** Effect of piR-hsa-211106 on PC mRNA levels (mean ± SEM, *p < 0.05); **(G)** Effect of piR-hsa-211106 on PC expression. Representative images of western blotting (left) and quantitative statistics (right) (mean ± SEM, *p < 0.05). All experiments were repeated three times. PC, pyruvate carboxylase.

In order to explore the mechanism by which piR-hsa-211106 regulates LUAD progression, we first used the miRBase database to predict miR-3180-3p as a potential binding microRNA of piR-hsa-211106. Subsequently, qRT-PCR was performed to assess the expression of miR-3180-3p, which was increased in A549 and HCC2279 LUAD cells (**Figure 5A**), but knocking down or overexpressing piR-hsa-211106 did not affect the expression of miR-3180-3p (**Figure 5B**), implying that piR-hsa-211106 does not play a regulatory role by binding to miR-3180-3p. Then, we performed RNA pull down to explore the proteins that bind to piR-hsa-211106 and then analyzed the pull-down proteins by LC-MS/MS. We obtained high-quality protein data according to requirements described in the *Materials and Methods* section, which contains twenty-six proteins including PC. Among these proteins, we selected seven candidates (PC, keratin 18, heat shock 70 kDa protein-8, lectin galactoside-binding soluble 3 binding protein, DNA-dependent protein kinase catalytic subunit, plakophilin-3, cortactin) that have been known to participate in lung cancer progression. However, overexpression or knockdown of piR-hsa-211106 only regulated the expression of PC, but not other proteins. Therefore, we focused our following investigation on PC (having five peptides: VVHSYEELEENYTR, AEAEAQAEELSFPR, AYVEANQMLGDLIK, QVGYEANGTVEFLGDR, DETATFGPLDSLNTR) (**Figure 5C**). Subsequent RNA pull-down and RIP assays confirmed interactions between piR-hsa-211106 and PC (**Figures 5D, E**). After overexpressing or knocking down piR-hsa-211106 at the cellular level, *PC* expression was inhibited or induced at the mRNA level (**Figure 5F**) and protein levels, respectively (**Figure 5G**).

### piR-hsa-211106 Can Enhance the Chemotherapy Sensitivity of Cisplatin

**Figure 6:**
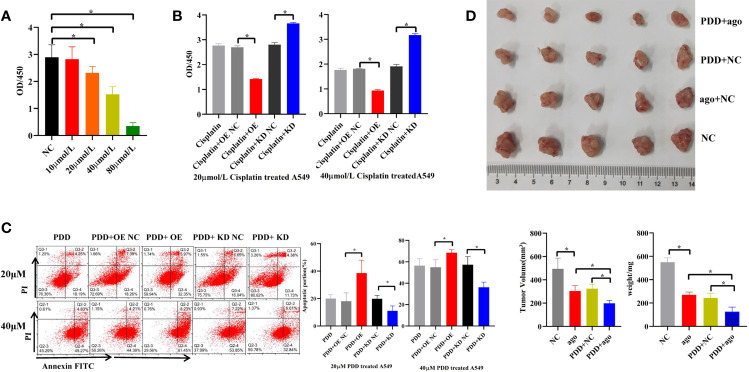
Effects of piR-hsa-211106 on the chemosensitivity of LUAD cells to cisplatin. **(A)** Effect of different concentrations of PDD on A549 cell proliferation after 96 h by CCK8 assay (mean ± SEM, *p < 0.05); **(B)** Effects of piR-hsa-211106 OE or KD on proliferation of LUAD cells induced by PDD tested by CCK8 assay (mean ± SEM, *p < 0.05). **(C)** Effects of piR-hsa-211106 OE or KD on apoptosis of LUAD cells induced by PDD based on flow cytometry. Representative images (left) and quantitative statistics (right) (mean ± SEM, *p < 0.05). **(D)** Injection of agopiR-hsa-211106 into xenograft tumors suppressed tumor growth in nude mice. Representative images (upper) and quantitative statistics (bottom right) (mean ± SEM, *p < 0.05). PDD, cisplatin; ago, agomir.

Different concentrations of cisplatin (PDD) (10, 20, 40, 80 μmol/L) were used to treat A549 cells *in vitro* for 96 h. Compared with the control, it was found that the proliferation activity of A549 cells decreased by 20, 40, and 80 μmol/L in a concentration-dependent manner (**Figure 6A**), indicating that PDD retards the proliferation of A549 cells *in vitro*. Subsequently, when cells were treated with 20 and 40 μmol/L PDD, we overexpressed or knocked down piR-hsa-211106 simultaneously. **Figures 6B, C** show that overexpression of piR-hsa-211106 acted synergistically with PDD to inhibit A549 cell proliferation and promotes cell apoptosis, while knocking down piR-hsa -211106 reversed this inhibitory effect of PDD on A549 cells. Then we confirmed the anti-cancer ability of piR-hsa-211106 *in vivo*. Compared with control, treated with piR-agomir could inhibit growth of xenografts, and enhance PDD treatment efficiency (**Figure 6D**). The above results show that piR-hsa-211106 can enhance the chemotherapy sensitivity of PDD.

## Discussion

This is the first study to identify a novel piRNA, piR-hsa-211106, which plays a critical role in LUAD as a tumor-suppressor RNA, and we further elucidated the novel regulatory mechanism underlying by which piRNAs regulate cancer. Overexpressed piR-hsa-211106 functions as an essential mediator that hinders PC signaling, consequently inhibiting proliferation and migration as well as promoting apoptosis of LUAD cells *in vitro*. Moreover, piR-hsa-211106 restricted the growth of mice xenograft models growth *in vivo*. Furthermore, we have demonstrated in mice xenograft models that treatment with piR-hsa-211106 agomir effectively suppressed tumor growth. piR-hsa-211106 was correlated with sensitivity to chemotherapy. piR-hsa-211106 enhanced the chemotherapy sensitivity of PDD *in vitro* and *in vivo*, implying that piR-hsa-211106 had synergistic anticancer effects with the chemotherapeutic agents of LUAD cells. Together, these findings provide new light insights into the role of piRNAs in the development and progression of human cancer and highlight the potential use of certain piRNAs as treatment targets for cancer.

Although piRNAs were initially identified in a mammalian germline, recent studies have shown that some of these piRNAs are also specifically expressed in other somatic tissues and aberrantly expressed in various cancer. Dysregulated piRNAs in tumor tissues have been revealed to play tumor-promoting or tumor-suppressor roles and are correlated with the tumor cell malignant phenotype and clinical stage (23, 24, 26). It has been suggested that certain piRNAs are involved in tumorigenesis through epigenetic mechanisms, such as causing DNA methylation at CpG sites in genome (27, 28). Another study showed that piRNAs bind to the transcriptional start site of the target gene, resulting in increased H3K4me3 but decreased H3K27me3 levels and activation of the gene (29). Otherwise, piRNAs could interact with other proteins, subsequently, alter their subcellular localization and facilitate the interaction of multiple proteins. For example, piR-L-163 interacts with phosphorylated ezrin-radixin-moesin proteins, resulting in accelerated DNA synthesis and G2-M cell cycle accumulation in human lung cancer cell lines (30). piR-54265 accelerates colorectal cancer proliferation and causes therapy resistance to anti-tumor agents by influencing STAT3 phosphorylation (31). Besides, piRNAs also could bind to and degrade its targeted RNAs by sequence complementary at post-transcriptional level. For instance, piR-55490 inhibits lung cancer cells and tumor proliferation by binding to the 3′ UTR of mTOR mRNA and degrading its targeted mTOR mRNA (32). piR-36712 suppresses breast cancer cell proliferation, invasion, and migration by combining with SEPW1P RNA (33). Above studies indicate that piRNAs play a vital role in various pathological processes.

In this study, we selected 10 most differently expressed piRNAs in LUAD tissues by high-throughput piRNA sequencing and confirmed their expression levels in our sample sets. Among the 10 piRNAs, only piR-hsa-211106 was significantly reduced in LUAD tissues compared with normal tissues, suggesting that piR-hsa-211106 may play an anti-oncogenic role in a LUAD tissue-specific manner. Afterward, we investigated the biological functions of piR-hsa-211106 *in vivo* and *in vitro*, and demonstrated that piR-hsa-211106 could inhibit *PC* both at the mRNA and at protein levels, as well as directly interacting with the PC protein. Katherine Sellers et al. have found that activity and expression of PC are enhanced in the early stages of human NSCLC development. Knock-down PC attenuated the proliferation and colony formation of A549 cell *in vitro* as well as suppressing tumor xenografts growth *in vivo* (13). High PC satisfies increased anabolic and energy demands of cancer cells and facilitates rapid cancer cells proliferation (34, 35). In our study, we identified that piR-hsa-211106 was as an upstream regulator of PC. Moreover, enforced piR-hsa-211106 was able to suppress the expression of PC at mRNA and protein level. Overexpression of piR-hsa-211106 reduced PC expression. It is known that PC suppression was accompanied by a decrease in anaplerotic input into the Krebs cycle, leading to reduction of nucleotide and lipid biosynthesis. Furthermore, PC inhibition in A549 cells greatly depletes the GSH pool and perturbs glutathione homeostasis, resulting in ROS production and a compromised ability to survive oxidative stress (13). Therefore, PC plays a key role in the piR-hsa-211106 inhibited LUAD progression. We hypothesize that piR-hsa-211106 might work in two ways: 1) piR-hsa-211106 in nuclear triggers PC gene methylation through DNA methylation pathways, thereby restricting the transcription and translation of PC gene; 2) piR-hsa-211106 in the cytoplasm combines with PC, hindering its normal function, thereby suppressing tumor growth. This novel function of piR-hsa-211106 in LUAD cells might serve as an example for studies on piRNAs and human cancers. However, the detailed mechanism by which piR-hsa-211106 regulates PC in LUAD progression remains to be addressed in the future.

Resistance to chemotherapy is also the main cause of cancer relapse and death of patients with cancer. However, the underlying mechanism has not been fully elucidated. Here, we determined that piR-hsa-211106 is involved in the drug-sensitivity of LUAD cells. We found that LUAD cells with piR-hsa-211106 overexpressing were sensitive, while those with piR-hsa-211106 knocked down were resistant to PDD, which is the first-line drug for chemotherapy of advanced LUAD (36). The underlying mechanism seems to be the pro-apoptotic role of piR-hsa-211106. Furthermore, we observed that piR-hsa-211106 synergistically acts with PDD both *in vitro* and *in vivo*. Because systemic chemotherapy has little effect in some cases, effective local therapy is often proposed as the next treatment regimen. In the present study, we also observed that direct injection of piR-hsa-211106 agomir into transplanted tumors in mice significantly inhibited tumor growth, indicating that piR-hsa-211106 agomir might be an effective drug for the treatment of LUAD.

## Conclusions

In the current study, we have identified piR-hsa-211106 as a tumor suppressor that is downregulated in LUAD and its underlying mechanism, providing evidence that piR-hsa-211106 has potential clinical value in the cancer development and treatment of LUAD patients.

## Data Availability Statement

The original contributions presented in the study are included in the article/**Supplementary Material**. Further inquiries can be directed to the corresponding author.

## Ethics Statement

The studies involving human participants were reviewed and approved by Ethics Committee of the Affiliated Hospital of Qingdao University. The patients/participants provided their written informed consent to participate in this study. The animal study was reviewed and approved by Ethics Committee of the Affiliated Hospital of Qingdao University.

## Author Contributions

YL conceived and designed the experiments, performed the experiments, formal analysis, wrote the paper. YD performed the experiments, reviewed the paper. XHe performed the experiments. AG performed the experiments. JG performed formal analysis. XHao performed formal analysis. SW collected and prepared the related literature. YF collected and prepared the related literature. ZW collected and prepared the related literature. ML collected and prepared the related literature. WX conceived and designed the experiments, contributed reagents/materials/analysis tools, reviewed the paper. All authors contributed to the article and approved the submitted version.

## Funding

This study was financially supported by the National Natural Science Foundation of China (81770900), the Qingdao Science and Technology Key Project (20-3-4-43-nsh); Major State Basic Research Development Program of Natural Science Foundation of Shandong Province in China (ZR2020ZD11); Science and Technology Key Project of Shandong Province (2014GHY115025); Science and Technology Research Project of Qingdao Science and Technology Bureau (16-6-2-28-NSH).

## Conflict of Interest

The authors declare that the research was conducted in the absence of any commercial or financial relationships that could be construed as a potential conflict of interest.

## Abbreviations

CPM, counts per million mapped reads; EdU, 5-ethynyl-2′-deoxyuridine; FISH, fluorescence *in situ* hybridization; LC-MS/MS, liquid chromatography-tandem mass spectrometry; LncRNA, long non-coding RNA; LUAD, lung adenocarcinoma; ncRNA, non-coding RNA; miRNA, micro-RNA; NC, negative control; nt, nucleotides; NSCLC, non-small cell lung cancer; OXPHOS, oxidative phosphorylation; PC, pyruvate carboxylase; PDD, cisplatin; piRNA, PIWI-interacting RNAs; qRT-PCR, quantitative real-time PCR; RIP, RNA immunoprecipitation; RT, reverse transcription; shRNA, short hairpin RNA.
