# Supplementary material for: piR-hsa-211106 Inhibits the Progression of Lung Adenocarcinoma Through Pyruvate Carboxylase and Enhances Chemotherapy Sensitivity
Source: Front Oncol. 2021 Jun 23;11:651915. doi: 10.3389/fonc.2021.651915 (PMC8260943; doi:10.3389/fonc.2021.651915)
Supplement: Supplementary file 1 [file Table_1.docx]

**Table S1 The proteins identified by the LC-MS/MS**

| **No.** | **ProteinName** | **PepCount** | **UniquePepCount** |
| --- | --- | --- | --- |
| 1 | Pyruvate carboxylase | 61 | 45 |
| 2 | Keratin 1 | 88 | 45 |
| 3 | Acetyl-CoA carboxylase 2 | 41 | 36 |
| 4 | Testicular secretory protein Li 29 | 33 | 24 |
| 5 | Autoantigen La | 34 | 26 |
| 6 | Lupus La protein | 34 | 26 |
| 7 | Methylcrotonoyl-CoA carboxylase | 33 | 24 |
| 8 | Propionyl-CoA carboxylase | 33 | 22 |
| 9 | Ataxin-2-like protein | 20 | 19 |
| 10 | Tubulin | 22 | 19 |
| 11 | keratin 18 | 19 | 17 |
| 12 | Actin | 19 | 16 |
| 13 | Nuclear fragile X mental retardation-interacting protein 2 | 17 | 15 |
| 14 | Polyadenylate-binding protein | 16 | 15 |
| 15 | Heterogeneous nuclear ribonucleoprotein U-like protein 1 | 14 | 14 |
| 16 | ATP-dependent RNA helicase DDX3X | 14 | 14 |
| 17 | RNA-binding protein 14 | 14 | 13 |
| 18 | RNA binding motif protein 14 isoform 1 | 14 | 13 |
| 19 | Transferrin | 12 | 12 |
| 20 | heat shock 70kDa protein-8 | 12 | 12 |
| 21 | lectin galactoside-binding soluble 3 binding protein | 14 | 12 |
| 22 | Galectin-3-binding protein | 14 | 12 |
| 23 | DNA-dependent protein kinase catalytic subunit | 11 | 11 |
| 24 | plakophilin-3 | 11 | 11 |
| 25 | cortactin | 11 | 11 |
| 26 | SNW1 protein | 10 | 10 |
